# Effectiveness of pericapsular nerve group block for hip fracture pain management in the emergency department: results of the ED-PENG-B randomised controlled trial

**DOI:** 10.1186/s12873-025-01401-x

**Published:** 2025-11-26

**Authors:** Patrick Calati, Camille Lenoir, Larbi Chaht Kamel, Nicolas Contie, Jean-Denis Firoloni, Adele Sichez, Annas Sebai, Jonathan Chelly, Laurent Caumon

**Affiliations:** 1 Emergency Medicine Department, Centre Hospitalier de Hyères, Hyères, France; 2 Clinical Research Unit, Délégation à la Recherche Clinique et à l’Innovation du Groupement Hospitalier de Territoire du Var, Centre Hospitalier Intercommunal Toulon La Seyne sur Mer, Toulon, France

**Keywords:** Hip fracture, Loco-regional anaesthesia, Pericapsular nerve group block, Pain, Emergency department

## Abstract

**Background:**

Hip fractures (HFs) managed in the emergency department (ED) are associated with severe pain. Locoregional anaesthesia (LRA) using the pericapsular nerve group (PENG) block may be an effective option for pain management in the ED, helping to reduce morphine use and improve postoperative rehabilitation.

**Methods:**

Patients admitted to the ED of a French tertiary hospital with suspected HF were enrolled and randomised into two groups: a standard of care (SOC) group receiving systemic analgesia in line with current recommendations, and an experimental group receiving systemic analgesia plus a PENG block. The primary outcome was morphine consumption per hour from randomisation until 24 hours post-randomisation or until surgery, if performed earlier.

**Results:**

Among the 35 randomised patients, 32 were included in the final analysis (11 men and 21 women; median age of 81 [74–91] years). Median morphine consumption per hour was significantly lower in the PENG group compared to the SOC group (0.2 [0.0–0.5] mg vs 0.4 [0.3–0.8] mg, respectively; *p* = 0.03). No significant differences were observed between groups in terms of total morphine use, pain scores (numeric rating scale), adverse events, or ED length of stay.

**Conclusion:**

Early PENG block appears to be a feasible and safe LRA technique when performed by trained emergency physicians and may reduce opioids requirements in patients with HF in the ED. Larger, adequately powered studies are warranted to confirm these findings.

**Trial registration:**

The study was registered prospectively at https://www.clinicaltrials.gov/ on 5 January 2023 (NCT05673486).

**Supplementary information:**

The online version contains supplementary material available at 10.1186/s12873-025-01401-x.

## Background

Hip fractures (HFs) are a frequent and growing reason for emergency department (ED) presentations. They predominantly affect frail elderly individuals (≥65 years) and are associated with considerable morbidity, mortality, and economic burden on healthcare systems. While opioids remain a cornerstone of acute pain management, their efficacy is inconsistent, and their use carries a risk of significant adverse effects, including delirium and falls [1–5].

Locoregional anaesthesia (LRA) techniques have been proposed and are now recommended, alongside standard analgesics, for managing HF-related pain in the ED [6–9]. Peripheral nerve blocks are increasingly employed to limit opioids use during acute care of HF patients [10]. Among these, the pericapsular nerve group (PENG) block has been introduced as a targeted method to anaesthetise the articular branches of the femoral and accessory obturator nerves, minimising motor block. PENG block has shown promise in preserving lower limb mobility postoperatively, thereby improving functional outcomes [11–13]. Despite demonstrated benefits in perioperative settings, the use of PENG block remains limited in EDs, and few studies have explored its efficacy in this context [14–19].

This study aimed to assess the efficacy of early PENG block administered in the ED by trained emergency physicians, in addition to standard intravenous (IV) analgesia, to reduce morphine use and improve pain control in patients presenting with HF.

## Methods

### Settings and patients

This open-label, single-centre, randomised controlled trial was conducted in the ED of a French tertiary hospital between April and December 2023.

Consecutive adult patients presenting to the ED with suspected HF and a numeric rating scale (NRS) pain score ≥3 were considered eligible. Patients were exclusively approached and screened after their admission to the ED. All patients were initially examined by both a nurse and a physician, as the entire ED team had been trained in the study protocol. The study was then presented to eligible patients, and informed consent obtained, exclusively by an investigator emergency physician specifically trained in performing the PENG block. To optimise management and radiological investigation, randomisation and treatment initiation were permitted upon clinical suspicion of HF. Patients without radiological confirmation were subsequently excluded. Exclusion criteria included age under 18 years, pregnancy, absence of pain, contraindication to the PENG block (e.g. Significant coagulation disorders, body mass index > 40 kg/m^2^, infection of lymphadenopathy at puncture site, allergy to local anaesthetics), or inability to provide informed consent.

This study was conducted according to the principles of the Declaration of Helsinki, received ethical approval (*Comité de Protection des Personnes Nord-Ouest IV*, Lille, France, approval number 22.04115.000146), and was registered prospectively at http://www.clinicaltrials.gov on 5 January 2023 (NCT05673486). Written informed consent was obtained from all participants or an independent witness when physical limitations prevented written signature. The study was sponsored by the *Centre Hospitalier Intercommunal de Toulon La Seyne-sur-Mer* and supported by the APICIL Foundation (Lyon, France). This trial follows the CONSORT guidelines for reporting randomised trials [20].

### Study protocol

Patients were randomised in a 1:1 ratio to receive either standard IV analgesia (standard of care, SOC group) or SOC plus PENG block (PENG group; see the experimental plan in Supplemental online content − 1). Randomisation used a permuted block design with variable block sizes, generated by an independent statistician using R Statistical Software (R Core Team 2021), and implemented via electronic case report forms. As shown in Supplemental online content − 2, all patients initially received 1000 mg IV paracetamol before inclusion; oral nefopam (20 mg) was permitted in patients < 75 years of age as needed. After randomisation, patients of the SOC groups received a weight-based IV morphine bolus, with titration every 5 minutes until a NRS < 3. The PENG group received the block as early as possible, and an optional weight-based IV morphine bolus was also allowed at the discretion of the attending physician, before considering morphine titration. Pain was then monitored hourly in the ED and every 4 hours afterwards. Paracetamol was repeated every 8 hours. Additional analgesics (non-steroidal anti-inflammatory drugs, nefopam, hip traction, ice pack, etc.) were permitted at the physician’s discretion. Follow-up continued until 24 hours post-randomisation or until surgery, whichever occurred first. Patients transferred to another hospital were followed until ED discharge.

### PENG block modalities

The PENG block was performed by the emergency physician under ultrasound guidance, targeting the area beneath the iliopsoas tendon near the inguinal ligament (see Supplemental online content − 3), as already described [10, 11]. A 20 ml dose of 0.5% ropivacaine was used, expected to provide analgesia for 12–24 hours. Physicians and nurses underwent a structured training programme comprising e-learning, a knowledge assessment, practical simulation, and at least one supervised procedure prior to study initiation.

### Study outcomes

The primary outcome was morphine consumption per hour (mg/h) from randomisation to 24 hours or until surgery, if performed earlier. Secondary outcomes included total morphine dose, changes in NRS (or ΔNRS) from inclusion to 24 hours and to ED discharge, maximum NRS during ED stay, adverse events, and ED length of stay. Opioids doses were standardised using a morphine-equivalent scale (see Supplemental online content − 4).

### Statistical analysis

Sample size estimation was based on the study by Tsai et al. [21], which compared femoral nerve block to SOC analgesia in HFs patients. Although this study did not evaluate the PENG block specifically, it was considered the most comparable in terms of patient population, study design, and statistical approach. At the time of our protocol development, available PENG block studies presented substantial methodological and population differences, which precluded their use for deriving robust statistical assumptions. Estimated morphine consumption was 0.22 ± 0.22 mg/h (SOC) vs 0.04 ± 0.11 mg/h (PENG). Assuming an α risk of 5% and a power of 80%, a sample size of 16 patients per group was calculated to confirm our hypothesis. Accounting for a 10% exclusion rate, 36 participants ware required. Categorical variables were expressed as n (%) and compared using the chi-squared test. Continuous variables were described using their median [25–75 interquartile range] and were compared with the Mann-Whitney test. Missing data were not imputed in the analysis of categorical variables. Analyses were performed using SAS^®^ 9.4.

## Results

### Study population

**Fig. 1:**
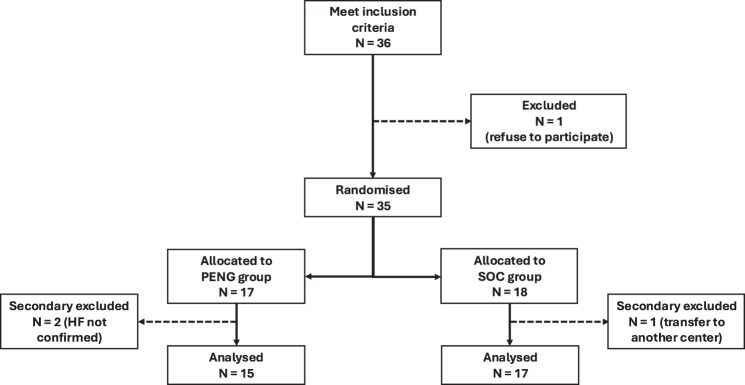
Flow chart of the ED-PENG-B trial (PENG: pericapsular nerve group; SOC: standard of care; HF: hip fracture)

Of the 36 eligible patients, 35 were randomised: 17 to the PENG group and 18 to the SOC group (one patient declined participation). Three patients were secondarily excluded (one for missing data, two for HF not confirmed radiologically), resulting in 32 patients analysed (15 in the PENG group and 17 in the SOC group) (Fig. 1).

Baseline characteristics are presented in Table 1. Median age was 81 years [74–91], with a male-to-female ratio of 1:2. No significant differences were found between groups regarding demographic, type of fracture, initial NRS scores, prior analgesic use, or time to first IV morphine dose. Vital signs were similar except for heart rate, which was slightly higher in the SOC group (90 [78–102] bpm vs 75 [72–91] bpm; *p* = 0.04). Twenty-four patients (75%) were admitted to a surgical ward, seven (21.9%) were transferred directly to the operating room, and one (3.1%) was hospitalised in the ED. Surgical treatment after ED discharge was performed in 8/15 patients (53.3%) in the PENG group and 6/17 (35.3%) in the SOC group.

**Table 1:** Baseline characteristics of the overall cohort and according to each allocation group

Characteristics	Overall cohort N = 32	SOC group N = 17	PENG group N = 15	p
**Age** – years	81 [74–91]	78 [72–89]	82 [75–94]	0.55
**Female sex**	21 (65.6)	10 (58.8)	11 (73.3)	0.38
**Body weight** – kg				
Median – kg	64 (15.1)	67(16.7)	63 (13.3)	0.5
< 60 kg ≥ 60 kg	12 (37.50) 20 (62.5)	5 (29.4) 12 (70.6)	7 (46.7) 8 (53.3)	0.31
**BMI** – kg/m^2^	24 [21–26]	25 [21–27]	23 [21–26]	0.52
**Hip fracture type**				
Femoral neck fracture	13 (40.6)	7 (41.2)	6 (40)	0.94
Trochanteric fracture	19 (59.4)	10 (58.8)	9 (60)	
**Analgesic before admission**	9 (28.1)	6 (35.3)	3 (20.0)	0.33
Paracetamol	7 (21.8)	5 (29.4)	2 (13.3)	
Aspirin	1 (3.1)	0 (0)	1 (6.7)	
Fentanyl	1 (3.1)	1 (5.9)	0 (0.0)	
Opioids	1 (3.1)	1 (5.9)	0 (0.0)	
Paracetamol/opium powder/caffeine	1 (3.1)	0 (0)	1 (6.7)	
**Analgesic at admission**				
Paracetamol	8 (25.0)	3 (17.7)	5 (33.3)	0.30
Nefopam	0 (0)	0 (0)	0 (0)	-
**Interval between admission and the first morphine dose** – min	135 [55–198]	117 [51–199]	142 [87–158]	0.71
**Interval between admission and X-ray** – min	104 [82–159]	99 [80–122]	110 [82–175]	0.70
**Vital parameters at admission**				
HR – bpm	87 [74–94]	90 [78–102]	75 [72–91]	0.04
RR – breath per min	18 [14–20]	18 [12–18]	18 [15–20]	0.20
SpO2 – %	96 [95–98]	96 [95–97]	97 [95–98]	0.42
MAP – mmHg	108 [99–115]	109 [104–125]	106 [91–114]	0.27
**Physician NRS assessment at ED admission**				
Median NRS	8 [5–9]	8 [5–9]	8 [5–10]	0.94
< 3	0 (0)	0 (0)	0 (0)	0.56
3–5	9 (28.13)	5 (29.41)	4 (26.67)	
6–8	12 (37.50)	5 (29.41)	7 (46.67)	
> 8	11 (34.38)	7 (41.18)	4 (26.67)	
**Interval between admission and randomisation** –min	42 [25–138]	96 [28–154]	35 [24–133]	0.49
Duration of participation * – hrs.	24 [16–24]	24 [15–24]	24 [17–24]	0.85
**Location after ED discharge**				
Surgery ward	24 (75.0)	14 (82.4)	10 (66.7)	0.43
Operating room	7 (22.0)	3 (17.6)	4 (26.7)	
ED ward	1 (3.0)	0 (0)	1 (6.6)	

Results are expressed as N (%) or median [interquartile range] unless otherwise specified. SOC: standard of care; PENG-B: pericapsular nerve group block; BMI: body mass index; ED: emergency department; HR: heart rate; RR: respiratory rate; MAP: mean arterial pressure; NRS: numeric rating scale. * Interval between randomisation and the end of participation to the study

### Study outcomes

**Fig. 2:**
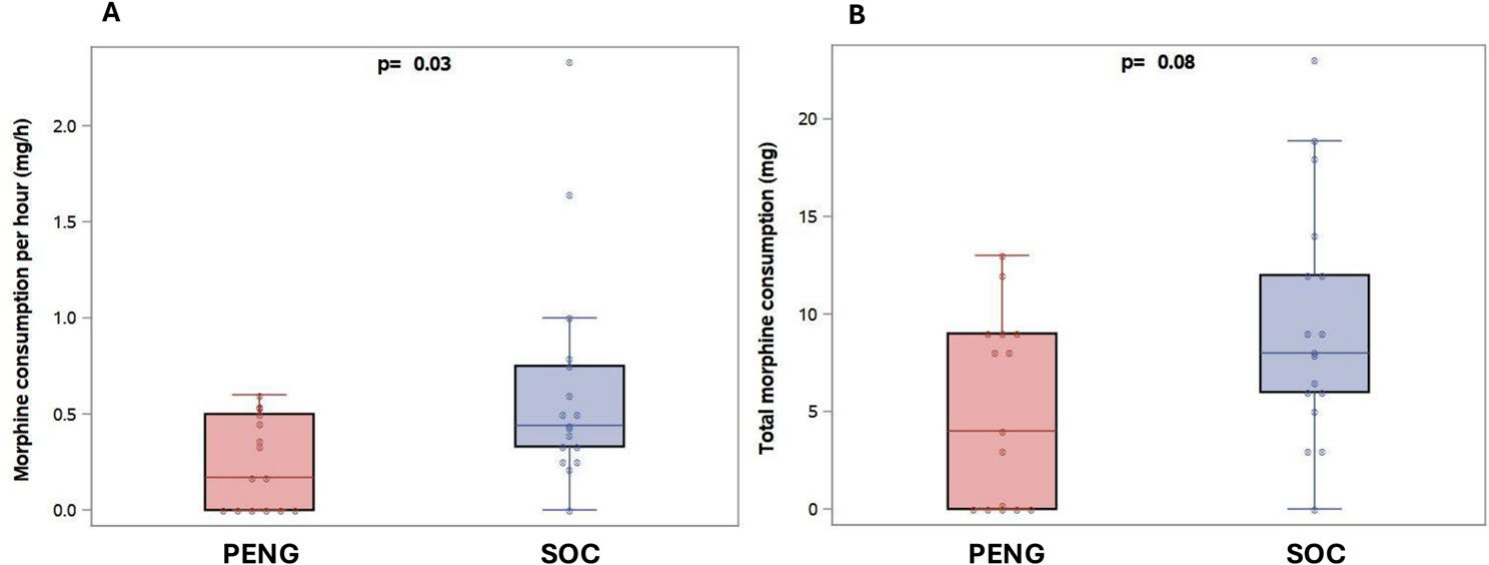
Box plots of morphine consumption per hour (panel A) and total morphine consumption (panel B), both from inclusion to emergency department discharge, according to each allocated group (PENG-B: pericapsular nerve group block; SOC: standard of care)

**Fig. 3:**
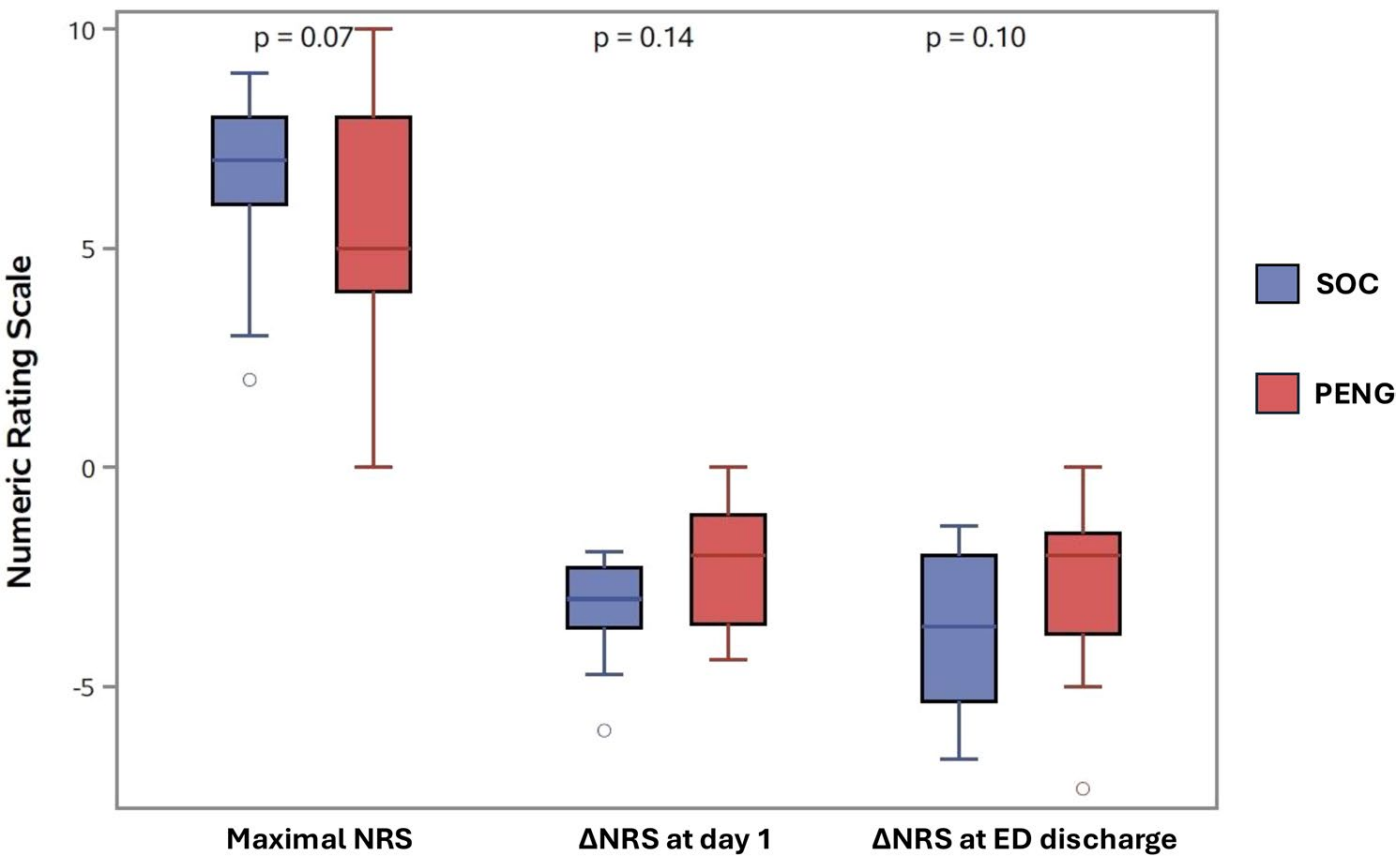
Box plots of maximal NRS, NRS variation from inclusion to day 1, and NRS variation from inclusion to ed discharge (NRS: visual analogue scale; ED: emergency department; PENG-B: pericapsular nerve group block; SOC: standard of care)

As shown in Table 2, median morphine consumption per hour was significantly lower in the PENG group compared with the SOC group (0.2 [0.0–0.5] mg/h vs 0.4 [0.3–0.8] mg/h; *p* = 0.03) (Fig. 2). No significant differences were observed for total morphine consumption (*p* = 0.08) (Fig. 2), ΔNRS at various time points (*p* = 0.07–0.14) (Fig. 3), adverse events (*p* = 0.38), or ED length of stay (*p* = 0.08). Additional analgesics used during the study period are detailed in the Supplemental online content − 5. No bleeding complications occurred in the PENG group (see the full list of the collected adverse events in the Supplemental online content − 6).

**Table 2:** Primary and secondary outcomes in the overall cohort and according to each allocated group

Outcome variables	Overall cohort N = 32	SOC group N = 17	PENG group N = 15	p
**Primary outcome**				
Morphine consumption per hour – mg *	0.4 [0.2–0.5]	0.4 [0.3–0.8]	0.2 [0.0–0.5]	0.03
**Secondary outcomes**				
Total morphine consumption – mg *	7.9 [1.5–9.0]	8.0 [6.0–12.0]	4.0 [0.0–9.0]	0.08
ΔNRS between inclusion and ED discharge	−3.0 [−1.7 – −4.5]	−3.6 [−2.0 – −5.3]	−2.0 [−1.5 – −3.8]	0.10
ΔNRS between inclusion and day 1	−2.9 [−1.9 – −3.6]	−3.0 [−2.3 – −3.7]	−2.0 [−1.1 – −3.6]	0.14
Maximal NRS **	6.5 [5.0–8.0]	7.0 [6.0–8.0]	5.0 [4.0–8.0]	0.07
Adverse event related to intervention	11 (34.4)	7 (41.2)	4 (26.7)	0.38
ED length of stay – hrs.	5.0 [3.0–7.5]	4.0 [3.0–6.0]	5.0 [4.0–18.0]	0.08

Results are expressed as N (%) or median [interquartile range] unless otherwise specified. SOC: standard of care; PENG: pericapsular nerve group; NRS: numeric rating scale; ΔNRS: the evolution of the NRS; ED: emergency department

* Between randomization and ED discharge

** Between randomization and the first 24 hours

## Discussion

This study is the first randomised trial assessing the impact of early PENG block performed in the ED by trained emergency physicians on morphine consumption in patients with HF. Our key findings are: 1) PENG block significantly reduced hourly IV morphine consumption during ED stay; 2) lower pain scores were observed in the PENG group, although not statistically significant; 3) there was no increase in adverse events or ED length of stay.

Previous studies have demonstrated the efficacy of PENG block for perioperative pain control and opioids reduction [21–26]. Although LRA has been advocated for ED use, data regarding PENG block specifically remain limited. Fahey et al reported safety in 52 patients receiving LRA in the ED, included 19 treated with the PENG block [27]. Güllüpinar et al randomised 39 patients to receive usual analgesics (paracetamol and tramadol) with or without PENG block and demonstrated pain reduction in the PENG group [18]. Although we did not replicate these results in terms of pain scores, our patients received a multimodal analgesic regimen, and the study was likely underpowered for this outcome. However, both studies confirmed reduced opioids use. Notably, opioids reduction observed with the PENG block contrasts with findings for other LRA techniques such as femoral nerve block [8, 21]. Furthermore, despite adding the PENG block procedure, no delays in management or ED length of stay were observed compared to the SOC group. The choice of hourly morphine consumption as the primary outcome aimed to standardize for variable ED length of stay and thereby reduce time-related confounding. Hourly morphine consumption was selected as the primary outcome to standardise for variable exposure time, since surgical timing in HF patients is highly variable: guidelines often target intervention within 24 h, yet in real practice delays may extend from a few to many hours depending on hospital logistics and patient optimisation [28]. In our data, total morphine consumption showed a non-significant trend favouring the PENG group, despite a tendency toward longer ED stays in that group. This pattern suggests a true reduction in the rate of opioids requirements after PENG that may not necessarily yield a significant difference in cumulative dose when exposure time differs, and the study is underpowered for secondary endpoints. These findings align with prior reports of opioids-sparing effects of PENG in perioperative and ED contexts [18, 22–27], while contrasting results have been described for other locoregional techniques such as femoral nerve block [8, 21]. Future adequately powered, multicentre trials should evaluate both rate- and time-standardised opioids outcomes alongside total consumption.

### Limitations

Several limitations must be acknowledged. First, the open-label design without placebo could have influenced management and pain reporting. However, placebo injections were deemed unethical given the established safety and efficacy of LRA techniques [21–25, 27], and placebo-controlled nerve block studies remain controversial [29]. Moreover, outcomes were not assessed by personnel blinded to the allocated treatment. Independent assessment would have required additional dedicated staff, which was not feasible given the workload in our ED. This lack of blinding may therefore represent a potential source of bias. Second, the monocentric design and small number of trained physicians limit generalisability. Third, pain was assessed using the NRS, a practical but imperfect tool [30], with irregular evaluation intervals complicating longitudinal pain assessment. However, it remains one of the most common and used scales for pain assessment. Fourth, pain during mobilisation was not specifically assessed, and the 24-hour study period limited outcome capture. However, PENG block benefits in elderly patients within this timeframe have been previously described in terms of pain management and postoperative cognitive functions [25]. Fifth, concerns may arise regarding the limited training programme of our study. However, no complication occurred, and the efficacy of the PENG block was confirmed by the result in our primary endpoint. Longer training and larger cohorts may however, further clarify potential benefits. Sixth, limiting follow-up to 24 hours might have missed delayed complications. However, experience with similar techniques suggests minimal risk (estimated incidence of long-term peripheral nerve injury: 2–4 per 10,000 blocks) [31]. Although motor preservation is less critical in HF than in arthroplasty patients, we believe that it facilitates comfort and in-bed mobilisation although awaiting surgery and helps surgeons assess functional status. Seventh, the power analysis was based on prior retrospective data with differences in population and anaesthetic agents used [21]. At the time of the study protocol design, few published data were available specifically evaluating the impact of early PENG block performed by emergency physicians. Regarding the choice of 80% power, this threshold was considered standard and appropriate for a monocentric study, especially given initial uncertainty about recruitment rates and available resources. A higher-powered study would strengthen validity but was limited here by feasibility. It is also important to highlight that, despite choosing this power threshold, we nevertheless achieved statistical significance for the primary outcome, suggesting that the observed effect size was sufficiently large to be detected within our sample. Another limitation is that the systematic use of an initial morphine bolus in the SOC group may have introduced bias in opioids consumption comparisons. However, this choice was made deliberately to reflect real-life ED practice and to ensure adequate analgesia for patients presenting with persistent pain on admission, while allowing discretionary use of the bolus in the PENG group. Importantly, the delay between inclusion and the first morphine bolus was similar in both groups, further limiting the risk of imbalance. We also acknowledge that prehospital care organisation varies across countries. In our local setting, patients with HFs generally do not receive opioids analgesia before hospital admission, but in other systems where such treatment is routinely administered, outcomes may differ, and this could limit the generalisability of our findings. Finally, our study should be regarded as a proof-of-concept trial. The relatively small sample size limited statistical power, prevented multivariate analysis, and led to minor baseline imbalances, thereby increasing the uncertainty of the results. Nevertheless, the consistent signal of reduced opioids use supports the feasibility of the intervention and provides a rationale for larger, adequately powered multicentre studies.

## Conclusions

Our findings suggest that early PENG block is a feasible and safe LRA technique when performed by trained emergency physicians and may reduce opioids use in patients with HF in the ED. Further adequately powered studies are required to confirm its impact on pain control. The PENG block represents a promising strategy for improving management in elderly patients with HF.

## Electronic supplementary material

Below is the link to the electronic supplementary material.

Supplementary Material 1

## Data Availability

Research data and other material (including the full protocol) will be made available to the scientific community, immediately upon publication, with as few restrictions as possible. All requests should be submitted to the corresponding author (Pr Jonathan Chelly) who will review with the other investigators for consideration. A data use agreement will be required before the release of participant data and institutional review board approval as appropriate.

## Abbreviations

ED: Emergency department
HF: Hip fracture
IV: Intravenous
LRA: Locoregional anaesthesia
NRS: Numeric rating scale
PENG: Pericapsular nerve group
SOC: Standard of care

## Declarations

### Patient and public involvement

Patients and/or the public were not involved in the design, or conduct, or reporting, or dissemination plans of this research.
