# Supplementary material for: Effectiveness of pericapsular nerve group block for hip fracture pain management in the emergency department: results of the ED-PENG-B randomised controlled trial
Source: BMC Emerg Med. 2025 Nov 26;25:245. doi: 10.1186/s12873-025-01401-x (PMC12659107; doi:10.1186/s12873-025-01401-x)
Supplement: Supplementary file 1 — Supplementary Material 1 [file 12873_2025_1401_MOESM1_ESM.docx]

**Effectiveness of pericapsular nerve group block for hip fracture pain management in the emergency department: results of the ED-PENG-B randomised controlled trial.**

Patrick Calati, Camille Lenoir, Larbi Chaht Kamel, Nicolas Contie, Jean-Denis Firoloni, Adele Sichez, Annas Sebai, Jonathan Chelly, Laurent Caumon.

**SUPPLEMENTAL ONLINE CONTENT**

**Supplemental online content – 1.** Experimental plan of the ED-PENG-B trial (T: time; ED: emergency department; SOC: standard-of-care; PENG: pericapsular nerve group)

# **Supplemental online content – 2.** Pain management algorithm according to each group (ED: emergency department; NRS: numeric rating scale; PENG: pericapsular nerve group block; SOC: standard-of-care; IV: intravenous).

# ****

## **Supplemental online content – 3.** Sonoanatomy of the PENG block with the white line indicating the direction of the needle for injection (FA: femoral artery; FV: femoral vein; FN: femoral nerve; AIIS: anterior inferior iliac spine; IPE: iliopubic eminence). From Ben Aziz M, Mukhdomi J. Pericapsular nerve group block. *StatPearls* [Internet]. <https://www.ncbi.nlm.nih.gov/books/NBK567757/>

**Supplemental online content – 4.** Morphine equivalent for each opioid used during the study.

| **Opioid used** (per 1 mg) | **Morphine equivalent** (mg) |
| --- | --- |
| Opium powder | 0.02 |
| Codeine | 0.05 |
| Tramadol | 0.06 |
| Oxycodone | 0.5 |
| Fentanyl | 830 |

**Supplemental online content – 5.** Analgesics used during the study period, according to each allocated group.

| **Analgesics used during the study period** | **SOC**  **group**  N = 17 | **PENG**  **group**  N = 15 |
| --- | --- | --- |
| Paracetamol | 17 (100.0) | 14 (93.3) |
| Morphine | 16 (94.1) | 10 (66.7) |
| Hip traction | 4 (23.5) | 0 (0) |
| Nefopam | 1 (5.9) | 2 (13.3) |
| Non-steroidal anti-inflammatory drugs | 0 | 1 (6.7) |
| Acetylsalicylic acid | 0 | 1 (6.7) |
| Variable are expressed as n (%). SOC: standard-of-care; PENG: pericapsular nerve group block | | |

**Supplemental online content – 6.** Adverse event during the study period according to each allocated group.

| **Adverse event** | **SOC**  **group**  N = 17 | **PENG**  **group**  N = 15 |
| --- | --- | --- |
| **Related to morphine** | 9 (52.9) | 6 (40,0) |
| Delirium | 1 (5.9) | 1 (6.7) |
| Nausea | 2 (11.8) | 2 (13.3) |
| Vomiting | 2 (11.8) | 2 (13.3) |
| Bradypnea | 2 (11.8) | 1 (6.7) |
| Acute urinary retention | 1 (5.9) | 0 (0) |
| Vertigo and itching | 1 (5.9) | 0 (0) |
| **Related to PENG block** | NA | 0 (0) |
| Variable are expressed as n (%). SOC: standard-of-care; PENG: pericapsular nerve group block | | |
